# Spinal Tumors and Tumor-like Masses: Relevance of Initial Imaging, Karnofsky Performance Status, Age, Location, and Cord Edema

**DOI:** 10.25259/JCIS-24-2019

**Published:** 2019-05-24

**Authors:** Surendra Kumar Gupta, Sonal Gahlot, Richa Singh, Virendra Singh

**Affiliations:** 1 Department of Radiodiagnosis, MLN Medical College, Allahabad, Uttar Pradesh, India.; 2 Department of Community Medicine, MLN Medical College, Allahabad, Uttar Pradesh, India.; 3 Department of Radiotherapy, MLN Medical College, Allahabad, Uttar Pradesh, India.

**Keywords:** Spinal tumors, Imaging, KPS score, Age, Location, Cord edema

## Abstract

**Objective:**

The aim of this study is to know the relevance of initial imaging, Karnofsky performance status,age,location and cord edema for future score development for radiologists.

**Methods:**

The present study was carried out on total of 32 patients who visited SRN Hospital and Kriti scanning centre between August 2009 to February 2019. General and systemic examination was done. Finally KPS score was given to each patient in accordance with their clinical status. Magnetic resonance imaging was contemplated using scanner – Magnetom SP Vision; Siemens –Supercon 1.0 Tesla system.

**Results:**

The morphologic characterization and specific assessment of various tumors and tumor like masses of spine in view of MR imaging features and their relationship with clinical features have been analyzed with Chi square test which reveal: Age versus location – *χ*^2^ = 4.32; df = 4; *P* > 0.36 (NS), age versus cord edema – *χ*^2^ = 4.27; df = 3; *P* > 0.23 (NS), and location versus cord edema – *χ*^2^ = 2.67; df = 2; *P* > 0.26 (NS). Chi Square test shows there is no any statistically significant association between age and location; age and spinal cord edema, and location and spinal cord edema. Correlation between clinical aggressiveness (change in follow-up KPS) and MR imaging features could not be assessed in our study because majority of patients could not be evaluated after one month because of loss of follow-up.

**Conclusion:**

Poor KPS itself lead to movement during MRI and movement related artifacts affecting initial imaging,which was managed by cotton padded neck strap. KPS depends only on general condition one of the factor for outcome so for future score development age,location and cord edema together may be useful.

## INTRODUCTION

Magnetic resonance imaging (MRI), myelography, post-myelo-computed tomography, and bone scan are the mainstay of evaluation of tumors of the spine. In the extradural space, MRI is the most sensitive technique for the detection of neoplasms in the vertebrae. The MRI may be tailored specifically toward the question of spinal cord compression and signal changes and cord edema. Clinical aggressiveness of tumors increases at extreme of ages. Clinical aggressiveness also depends on tumor doubling time.

The Karnofsky performance status (KPS) scale has been used as an assessment tool for performance status in oncology since 1948.^[1]^ It is commonly regarded as the gold standard measurement of performance status in cancer.^[2,3]^ The KPS scale assesses three dimensions of health status – activity, work, and self-care and can be administered by any health-care professional for a quick assessment of general functioning and survival.^[4]^ When KPS is low, it is a sensitive predictor of poor prognosis, but when high it is a poor cross-sectional indicator of prognosis.^[2]^ KPS decrease by 20 points over a month time has been suggested as low clinical aggressiveness, 40 points decrease as moderate clinical aggressiveness, and 60 points decrease as high clinical aggressiveness.^[1]^ The aim of this study is to know the relevance of initial imaging, KPS, age, location, and cord edema for future score development for radiologists.

## MATERIALS AND METHODS

The present study was carried out on total of 32 patients visiting the Radiology Department of SRN Hospital of MLN Medical College, Allahabad, and Kriti Scanning Centre between August 2009 and February 2019.

Written consent was taken from the patients subjected to this study. A detailed history including back pain, weakness or paralysis, sensory loss, and change of bowel function was taken. General and systemic examination was done followed by neurological examination to localize the level of cord lesion. Finally, KPS score was given to each patient in accordance with their clinical status. Patient was sent for treatment and follow-up KPS was assessed after 1 month.

MRI was contemplated using scanner – Magnetom SP Vision; Siemens – Supercon 1 Tesla system, with only dedicated (body: L and XL for thoracic + lumbar spine and cervical spine) coils. T1W and T2W sequences in sagittal and axial plane were taken for thoracic and lumbar spine and sagittal T1W and T2W and axial T1 and gradient-recalled echo images for cervical spine as standard protocol.

In addition, short inversion time inversion recovery sequences to suppress fat in paraspinal soft tissue, FLASH, and T1W FAT-SAT sequences were also included wherever necessary.

Post contrast study was also carried out with intravenous Gadopenate Dimeglumine when needed.

## RESULTS

**Figure 1:**
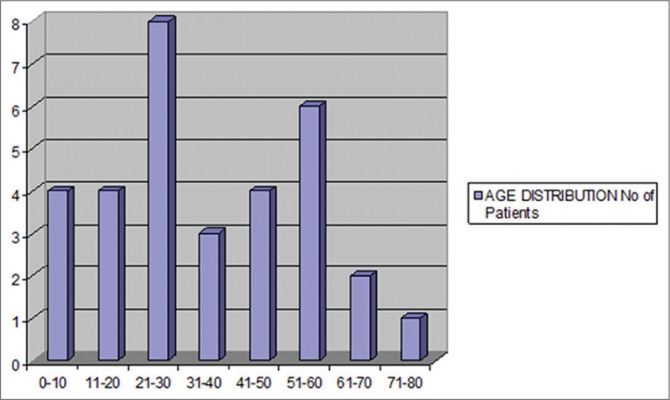
Age and sex wise distribution of patients.

A total of 32 patients were included in the study, 66% of affected were male and the most common age group involved was 21–40 years (34.47%) age group [Figure 1]. The only diagnosed entity in this age group was neoplastic disease which included – metastasis, cavernous hemangioma, astrocytoma, chordoma, lipoma, lymphoma, three cases of meningioma, and two cases of ependymoma. No tumor-like mass was seen.

Overall, the most common neoplasm was meningioma (15.6%) followed by lymphoma (12.5%).

The most common location involved was intradural extramedullary (37.93%) comprising meningioma, nerve sheath tumor, lipoma, epidermoid, and metastasis [Table 1]. 34.48% of cases were seen in extradural compartment and 27.59% of cases in intramedullary compartment. Maximum incidence of secondary neoplasms was seen in extradural compartment (60% of total secondary).

**Table 1:** Distribution of tumors and tumor-like masses based on location.

Location of tumor	Number of cases		
	Tumor (*n*=29)		Tumor-like mass (*n*=3)
	Primary	Secondary	
Extradural (*n*=11)			
Osseous	5	3	1
Paraspinal muscles	1	0	0
Epidural	1	0	0
Intradural extramedullary (*n*=11)	10	1	0
Intramedullary (*n*=10)	7	1	2
Total	24	5	3

**Figure 2:**
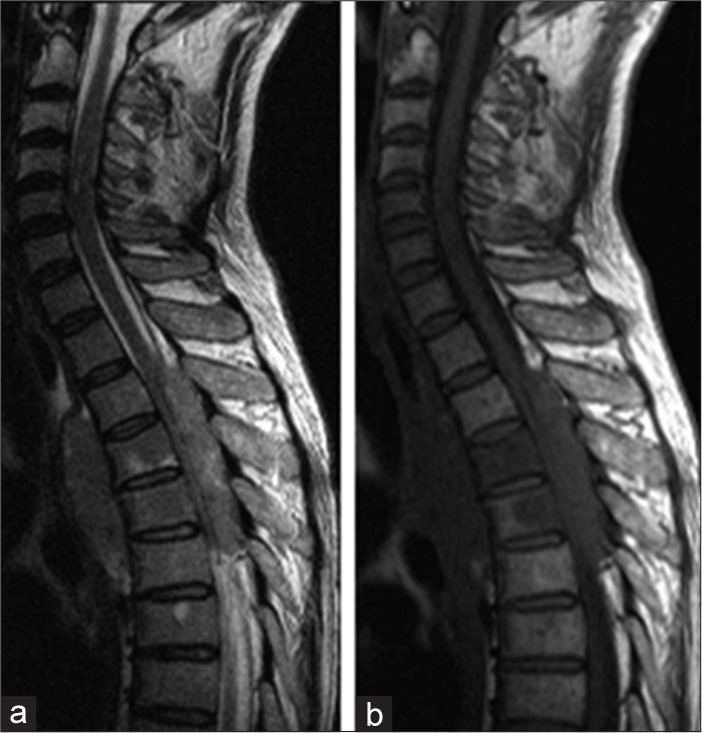
Metastasis: Know case of bronchogenic carcinoma presented with sudden onset quadriparesis: sagittal T2 W (a) images show multiple patchy hyperintense signal changes in D3 and D4 vertebral bodies with minimal loss of vertebral body height and presence of large abnormal prevertebral and posterior epidural hypointense soft tissue compressing narrowing spinal cord with foal cord edema.-Sagittal T1 W images (b) shows hypointense signal changes in D3 and D4 vertebral bodies with minimal loss of vertebral body height and presence of large abnormal prevertebral and posterior epidural hypointense soft tissue compressing narrowing spinal cord.

Of the neoplastic cause, secondary tumor, i.e., metastasis was accounting for 17.24% of cases. Extradural osseous location constitutes 60% of metastatic tumors [Figure 2]. Here, destruction of pedicles appears to occur only in combination with involvement of posterior portion of vertebral body.

**Figure 3:**
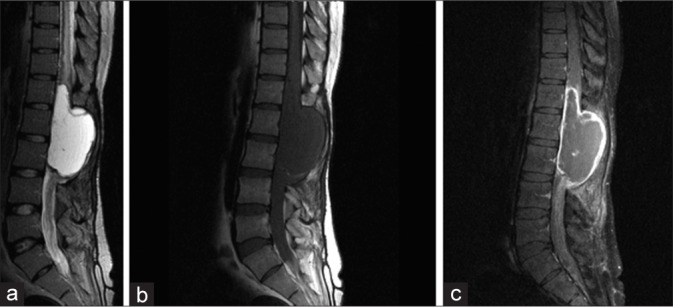
Ependymoma Conus medullaris: a) sagittal T2W image shows large well-defined septated intradural extramedullary hyperintense mass related to conus medullaris causing focal scalloping and widening of spinal canal and compressing narrowing conus with focal cord edema. (b) Sagittal T1W images show that lesion is hypointense on T1W images and shows peripheral T1 hyperintense rim suggestive of hemorrhage, (c) sagittal post-contrast fat-sat T1W images reveal peripheral enhancing rim of lesion with subtle patchy intralesional.

Among primary non-lymphoproliferative tumors (*n* = 22), the most common entity was meningioma (22.72%) followed by nerve sheath tumors (13.63%), astrocytoma (13.63%), and ependymoma [Figure 3] (13.63%). Besides, these other varieties noted were epidermoid cyst, intradural extramedullary lipoma, paraspinal soft tissue hemangioma, Ewing’s sarcoma, sacrococcygeal chordoma, and intramedullary hemangioma [Table 2].

**Table 2:** Compartment-wise distribution of various tumors and tumor-like masses.

Compartment	Tumor	No (%)	Tumor-like masses	No (%)
Intramedullary (*n*=8)	Ependymoma	3 (10.34)	Granuloma	1 (33.33)
	Astrocytoma	3 (10.34)	Syrinx	1 (33.33)
	Cavernous hemangioma	1 (3.44)		
	Metastasis	1 (3.44)		
Intradural extramedullary (*n*=11)	Meningioma	5 (17.34)		
	Nerve sheath tumor	3 (10.34)	None	0 (0)
	Lipoma	1		
	Epidermoid	1		
	Metastasis	1		
Extradural (*n*=10)	Lymphoma	4	Tubercular osteomyelitis	1 (33.33)
	Metastasis	3		
	Ewing’s sarcoma	1		
	Chordoma	1		
	Cavernous hemangioma	1		

The maximum incidence of spinal neoplasm was noted at dorsal spine (41.38%) followed by eight cases at dorsolumbar and lumbar spine (27.58%) in the present study.

**Figure 4:**
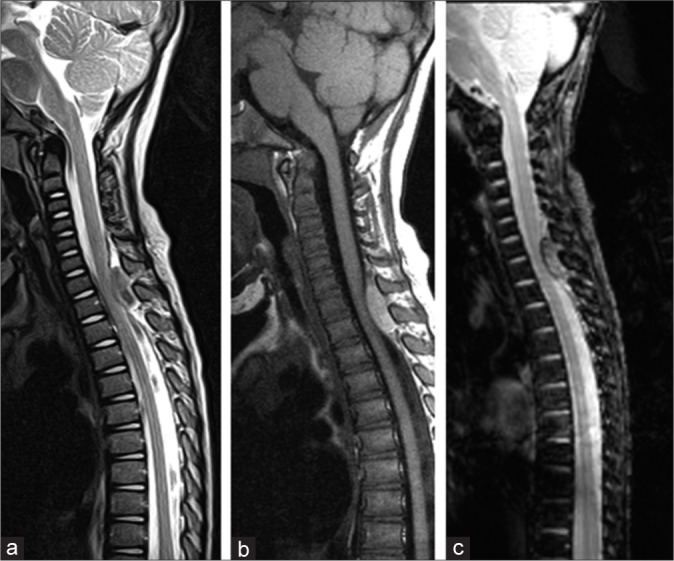
Epidural hematoma: (a) sagittal T2W image shows small posterior extradural hypointense soft tissue compressing narrowing spinal cord with focal cord edema. (b) Sagittal T1W image shows that lesion is homogeneously hyperintense on T1W images. (c) Sagittal T2 FLASH images reveal peripheral as well as central specks of blooming.

Four non-neoplastic tumor-like masses seen – (i) tuberculous spondylodiskitis (ii) intramedullary granuloma, (iii) syrinx, and (iv) epidural hematoma [Figure 4].

Evidence on MRI of involvement of two consequent vertebrae and the intervening disk is virtually diagnostic of infective spondylitis. In tuberculous spondylitis, the cortical definition of the affected vertebrae was found to be invariably lost, in contradistinction to pyogenic spondylitis.

In the present study, solitary cervical cord granuloma was seen. Intracranial tuberculomas have been described as low-intensity lesions with or without central hyperintensity (due to varying amount of caseous necrosis) on T2WI and as hypo to isointense lesions on T1WI. In the present study, same features were seen.

Syringohydromyelia was observed in association with dorsal dermal sinus. In the present study, cord edema was seen in 19 (59.4% of total) patients, of which 73.68% of patients have >70 initial KPS at the time of MRI, 21.05% have KPS 50–70, and 5.26% have <50 KPS. 61.35% of cases having no spinal cord edema, 13–40.6% of total patients have initial KPS >70, 30.78% have KPS 50–70, and 7.69% of cases have initial KPS <50. Thus, majority of patients of both groups, having cord edema present and absent, have good KPS that is >70 [Table 3]. Thus, there was no significant association between MRI features and initial KPS. Furthermore, no significant correlation was seen between clinical features and MRI features whether they are typical features of particular diagnosis or features are indeterminate.

**Table 3:** Distribution of tumors and tumor-like masses based on initial KPS values and presence or absence of edema.

Initial KPS	Cord edema present (*n*=19) (Number of patients)	Cord edema absent (*n*=13) (Number of patients)
>70	14 (73.68)	8 (61.53)
50–70	4 (21.05)	4 (30.78)
<50	1 (5.26)	1 (7.69)

KPS: Karnofsky performance status

In the present study, majority of patients of both groups, having cord edema present and absent, have good KPS that is >70. Furthermore, patients with MRI features of all three groups (typical/variable/indeterminate) had same distribution of KPS [Table 4].

**Table 4:** Initial KPS values and MRI features.

Initial KPS score	Typical MRI features (Number of patients)	Variable/additional finding (Number of patients)	Indeterminate (Number of patients)
>70	15	5	3
50–70	8	0	0
<50	0	1	0

MRI: Magnetic resonance imaging, KPS: Karnofsky performance status

The morphologic characterization and specific assessment of various tumors and tumor-like masses of spine in view of MRI features and their relationship with clinical features have been analyzed with Chi-square test which reveal: Age versus location – *χ*^2^ = 4.32; df = 4; *P* > 0.36 (NS), age versus cord edema – *χ*^2^ = 4.27; df = 3; *P* > 0.23 (NS), and Location versus cord edema – *χ*^2^ = 2.67; df = 2; *P* > 0.26 (NS). Chi-square test shows that there is no statistically significant association between age and location; age and spinal cord edema; and location and spinal cord edema.

KPS decrease by 20 points over a month time has been taken as low clinical aggressiveness, 40 points decrease as moderate clinical aggressiveness, and 60 points decrease as high clinical aggressiveness. Correlation between clinical aggressiveness (change in follow-up KPS) and MRI features could not be assessed in our study because majority of patients could not be evaluated after 1 month due to loss of follow-up [Table 5].

**Table 5:** KPS values initially and during follow-up in relation to MRI features.

MRI features	Initial KPS	Median initial KPS	KPS after 1 month	Median KPS after 1 month
Indeterminate	90 (*n*=1)	80	LTF	Cannot be calculated
	80 (*n*=2)		LTF	
Variable/additional (Internet literature search)	100 (*n*=1)	80	100	
	80 (*n*=3)		LTF	
	40 (*n*=1)		LTF	
	90 (*n*=1)		LTF	
Typical features	50 (*n*=2)	50	50	50
	60 (*n*=1)		60	
	50 (*n*=4)		LTF	
	60 (*n*=1)		LTF	
	80 (*n*=9)		LTF	
	90 (*n*=5)		LTF	
	100 (*n*=1)		LTF	

LTF: Lost to Follow-up, MRI: Magnetic resonance imaging, KPS: Karnofsky performance status

The primary tumor of spine may exhibit characteristic imaging features that can help in early diagnosis and improved prognosis.

## DISCUSSION

The present study focuses on the MRI evaluation in tumors and tumor-like masses of the spine and correlation between MRI features of disease and clinical aggressiveness. MRI has been contemplated as the initial procedure in the evaluation of 32 cases of clinically suspected spinal tumor.

According to Nittner, approximately one-fifth of all central nervous system tumors occur in the spine with frequencies at various level of spinal canal (cervical, thoracic, and lumbar) roughly proportional to the number (and length) of segments at that level.^[5,6]^ The same observation was quoted by Masaryk^[6]^ In the present study, the most common location is dorsal spine (44.83%) followed by lumbar (17.24%) and then cervical (13.79%) and dorsolumbar (10.34%) spine. Chordoma in our study also had predominant large soft tissue mass anterior to sacrum as noted in other studies.^[8]^

According to their anatomical location, spinal tumors are conveniently classified as extradural, intradural extramedullary, and intramedullary tumors with intradural extramedullary tumors being the most common (50%) followed by extradural tumors (30%) and least common were intramedullary tumors (20%).^[9]^ The present study has same distribution as above and following observation has been summarized below: Intradural extramedullary – 37.93%, Extradural – 34.48%, and intramedullary – 27.59%.

The primary tumor of spine may exhibit characteristic imaging features that can help in early diagnosis and improved prognosis.^[10]^

**Figure 5:**
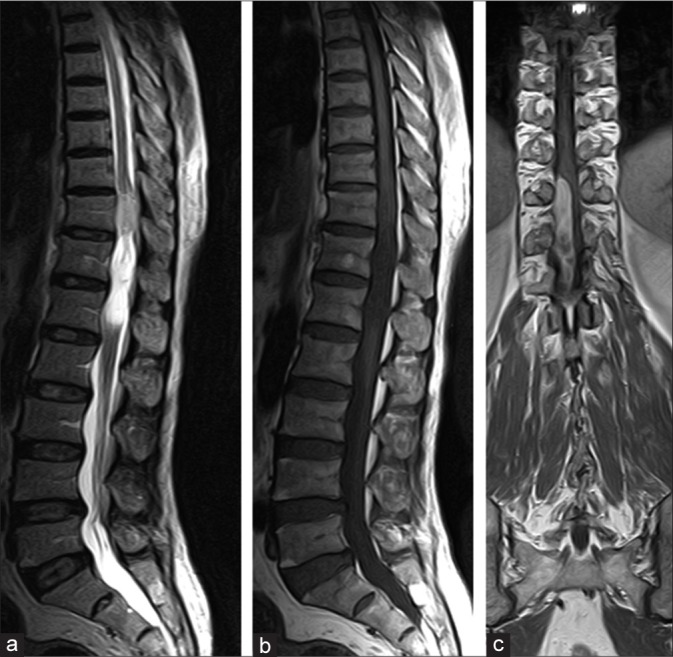
Nerve sheath tumor: (a and b) Sagittal T2 and T1W images show well-defined heterogeneous hyperintense on T2 and hypointense on T1 intradural extramedullary space occupying lesion with mild perifocal cord edema. (c) Post-gadolinium coronal T1W image shows heterogeneous enhancement of lesion lying on the right side with no neural foraminal extension.

Nittner in his 1976 review of 4885 adult spinal cord tumors presented in literature, found nerve sheath tumor [Figure 5] (23%), meningiomas (22%), glial tumors (13.2%), ependymomas (2.5%), sarcoma (8.2%), and metastasis (6%) to be the most common.^[7]^ The remaining 25% of cases were dispersed among a wide variety of miscellaneous mass lesions. In the present study, metastasis and meningioma account for the most common neoplasia (17.24% each), followed by nerve sheath tumor, astrocytoma, and ependymoma (10.34% each). The rest of the entities are lymphoma, lipoma, hemangioma, and chordoma.

Meningiomas are second only to nerve sheath tumors in frequency. However, in the present study, meningioma (17.24%) was more common than nerve sheath tumor (10.34%).

In the present study, from infective group of tumor-like masses, two cases were seen, one case seen of tubercular osteomyelitis and one of intramedullary granuloma.

Spinal tuberculosis can occur at any age and affects both sexes equally. Since dura constitutes an effective barrier against intraspinal spread of infection, extradural lesions are most common while intradural extramedullary lesions are least common.^[9]^ In the present study, one case was intramedullary granuloma.

## CONCLUSION

Poor KPS itself leads to movement during MRI and movement-related artifacts affect initial imaging interpretation, this was managed by cotton padded neck strap instead of the use of anesthesia so KPS has relevance to initial imaging interpretation. KPS depends on general condition, only one of the factors related to outcome so for future score development age, location, and cord edema together may be useful.

## APPENDICES

**Table:**

Score (Category)	KPS scale
100 (A)	Normal; no complaints; no evidence of disease
90 (A)	Able to carry on normal activity; minor signs or symptoms
80 (A)	Normal activity with effort; some signs or symptoms of disease
70 (B)	Cares for self; unable to carry on normal activity or to do active work
60 (B)	Requires occasional assistance but is able to care for most of his needs
50 (B)	Requires considerable assistance and frequent medical care
40 (C)	Disabled; requires special care and assistance
30 (C)	Severely disabled; hospitalization necessary; active supportive treatment is necessary
20 (C)	Very sick; hospitalization necessary; active supportive treatment is necessary
10 (C)	Moribund; fatal processes progressing rapidly
0	Dead

KPS: Karnofsky performance status
